# Traumatic Brain Injury in the Netherlands: Incidence, Costs and Disability-Adjusted Life Years

**DOI:** 10.1371/journal.pone.0110905

**Published:** 2014-10-24

**Authors:** Annemieke C. Scholten, Juanita A. Haagsma, Martien J. M. Panneman, Ed F. van Beeck, Suzanne Polinder

**Affiliations:** 1 Department of Public Health, Erasmus University Medical Center, Rotterdam, The Netherlands; 2 Research Department, Consumer and Safety Institute, Amsterdam, The Netherlands; University of South Florida, United States of America

## Abstract

**Objective:**

Traumatic brain injury (TBI) is a major cause of death and disability, leading to great personal suffering and huge costs to society. Integrated knowledge on epidemiology, economic consequences and disease burden of TBI is scarce but essential for optimizing healthcare policy and preventing TBI. This study aimed to estimate incidence, cost-of-illness and disability-adjusted life years (DALYs) of TBI in the Netherlands.

**Methods:**

This study included data on all TBI patients who were treated at an Emergency Department (ED - National Injury Surveillance System), hospitalized (National Medical Registration), or died due to their injuries in the Netherlands between 2010–2012. Direct healthcare costs and indirect costs were determined using the incidence-based Dutch Burden of Injury Model. Disease burden was assessed by calculating years of life lost (YLL) owing to premature death, years lived with disability (YLD) and DALYs. Incidence, costs and disease burden were stratified by age and gender.

**Results:**

TBI incidence was 213.6 per 100,000 person years. Total costs were €314.6 (USD $433.8) million per year and disease burden resulted in 171,200 DALYs (on average 7.1 DALYs per case). Men had highest mean costs per case (€19,540 versus €14,940), driven by indirect costs. 0–24-year-olds had high incidence and disease burden but low economic costs, whereas 25–64-year-olds had relatively low incidence but high economic costs. Patients aged 65+ had highest incidence, leading to considerable direct healthcare costs. 0–24-year-olds, men aged 25–64 years, traffic injury victims (especially bicyclists) and home and leisure injury victims (especially 0–5-year-old and elderly fallers) are identified as risk groups in TBI.

**Conclusions:**

The economic and health consequences of TBI are substantial. The integrated approach of assessing incidence, costs and disease burden enables detection of important risk groups in TBI, development of prevention programs that target these risk groups and assessment of the benefits of these programs.

## Introduction

Traumatic brain injury (TBI) – defined as an alteration in brain function, or other evidence of brain pathology, caused by an external cause [1] – is a leading cause of morbidity, disability, and mortality worldwide. In Europe, the annual incidence rate of hospitalized and fatal TBI is about 235 per 100,000 person years [2]. TBI survivors almost all experience some level of impairment or disability [2], which drastically reduces their health-related quality of life (HRQL) [3], [4].

In addition to the often long-term impact of TBI on a person's life, the economic consequences of TBI for both individuals and society are substantial [5], [6]. TBI patients require specialized pre-hospital care, transport, in-hospital (emergency) care, and often long-term rehabilitation. Survivors of more severe TBI are often unable to return to full employment [7], [8]. TBI therefore leads to significant direct healthcare costs in terms of pre-hospital care, emergency care, hospitalization, long-term outpatient care and rehabilitation, and indirect costs due to loss of productivity. The total direct and indirect costs of TBI occurring in Europe were estimated to €33 billion (approximately USD $45.4 billion) [9].

Most efforts on assessing the impact of TBI have been limited to either its epidemiology [2], [10], [11], costs [5], [6], [9], [12]–[18] or disease burden [17], [19], [20]. Integrated knowledge on epidemiology, economic consequences and disease burden of TBI is scarce but essential for optimizing healthcare policy, allocating scarce resources, preventing TBI, and developing effective healthcare and rehabilitation services. Up till now, an insight of the total population impact of TBI is lacking. The purpose of this study was to estimate the incidence, cost-of-illness and disability-adjusted life years (DALYs) of TBI in the Dutch population, and to detect important risk groups in TBI.

## Methods

### Data sources

This surveillance-based study included data on all patients with TBI treated at an ED and/or admitted to hospital in the Netherlands in the period 2010–2012. TBI cases were extracted from the Dutch Injury Surveillance System (LIS) [21] and the National Hospital Discharge Registry (LMR) [22], to include data of TBI patients treated at the ED and hospitalized TBI patients respectively.

LIS is an ongoing monitoring system which records data of all unintentional and intentional injured patients who attend the ED. LIS is based upon the registration of 13 hospitals in the Netherlands (12–15% coverage) that are considered to be representative for the total Dutch injury-related ED visits. To generate national estimates of the injury-related ED visits in the Netherlands, an extrapolation factor was calculated in which the number of ED treatments due to injury registered by the participating hospitals is multiplied by the quotient of the number of hospital admissions due to injury in the Netherlands divided by the number of hospital admissions due to injury registered in the participating hospitals [23]. The required data on the number of hospital admissions due to TBI in the Netherlands is obtained from the LMR, which collects data from all Dutch hospitals regarding patient information from hospital admission to discharge. In this study, data from LIS was used to assess socio-demographic (age at injury and sex), injury (type of injury, external cause of injury, multiple injury), and healthcare related characteristics (hospitalization and length of stay). To avoid double counting, only the LMR was used to obtain data of hospitalized patients on the type of injury (ICD-9-codes) and for costs calculations.

### Definition of TBI

For patients treated at the ED, TBI was defined as having a “Concussion” or “Other skull – brain injury” in at least one of the three injuries that can be recorded in LIS. This study therefore included all cases in which TBI was registered as first, second or third injury. In case of multiple injuries, an hierarchy derived from the literature was used to determine the most severe injuries [24]. This hierarchy prioritized spinal cord injury over skull or brain injury (except concussions), hip fracture, and other lower extremity fractures, respectively. For hospitalized patients, TBI was defined using the International Classification of Diseases, ninth revision (ICD-9-CM). This study included ICD-9-codes related to concussion (850), fractures (800–801, 803, 804), lesion (851–854), late effects (905, 907), nerve injury (950), and unspecified head injury (959).

### Cost-of-illness

Short- and long-term direct costs (e.g., healthcare costs) and indirect costs (e.g., productivity loss) of TBI were calculated with use of the incidence-based Dutch Burden of Injury Model [23], [25]. This model calculates patient numbers, healthcare consumption, and related costs for predefined patient groups that are homogenous in terms of health service use. Data on healthcare consumption was obtained from the LIS and LMR database, rehabilitation centers (LIVRE), nursing homes (SIVIS), and a patient follow-up survey conducted in 2007–2008 [23], [26], [27].

Direct healthcare costs of TBI were calculated by multiplying incidence by healthcare volumes (e.g., length of stay), transition probabilities (e.g., probability of hospital admission), and unit costs (e.g., costs per day in hospital). All unit costs were estimated according to national guidelines for healthcare costing [28], reflecting real resource use (Table 1). Indirect costs of TBI were calculated for all TBI patients in the working age 15 to 64 years treated at the ED or hospitalized, based on information on work absence and return to work from the patient follow-up questionnaire conducted in 2007–2008 [23], [26], [27].

**Table 1:** Unit costs (2012).

Resource	Unit costs
**General Practitioner**	
Practice consultation	€33.70
Consultation by telephone	€16.90
Home visit	€67.40
Referral patient treated at the ED	€35.00
Referral hospitalized patient	€44.00
Follow-up care patient treated at the ED	€33.70
Follow-up care hospitalized patient	€37.80
**Ambulance**	
Emergency journey	€538.20
Scheduled journey	€206.20
**Hospital**	
Attendance of emergency department	Injury specific fees^1^
Hospitalization general hospital	€460.40/day
Hospitalization academic hospital	€629.00/day
Intensive care	€1,751.50/day
Day care	€310.30/day
Outpatient department visit	€178.10/visit
Medical procedures	Reimbursement fees
**Long term care**	
Nursing home	€264.60/day, 138.80/day care
Rehabilitation	€469.10/day
Physiotherapy	€38.00/treatment
**Home care**	
Domestic care	€30.60/h
Care	€39.10/h
Nursing	€67.60/h
Nursing & care	€46.40/h
**Labor costs (including VAT)**	
15–19 year	€13.50/hour
20–24 year	€24.70/hour
25–29 year	€32.80/hour
30–34 year	€39.30/hour
35–39 year	€43.30/hour
40–44 year	€45.40/hour
45–49 year	€46.80/hour
50–54 year	€48.50/hour
55–59 year	€49.70/hour
60–64 year	€50.70/hour
Overall mean	€40.90/hour

1 Unit costs for attendance of emergency department are calculated per type of injury in an annually unit cost study indexing the tariffs per minute of nurses, physicians and specialists.

ED: emergency department; VAT: value added tax.

In order to compare the costs of TBI in the Netherlands with previous cost studies conducted in other countries and at varying points in time, all costs estimates were adjusted for inflation with use of the Consumer Price Index [29]– and converted into 2012 Euros (as at 31 December 2012 €1.00 =  USD $1.3203).

### Burden of TBI

The national disease burden of TBI was measured using the disability-adjusted life year (DALY), a summary measure of population health [32]. To calculate the burden of disease, information on premature mortality, and morbidity and disability due to non-fatal health outcome is combined into one single number. This number represents the health gap between the current state of a population's health compared to an ideal situation where individuals would live to the standard life expectancy in full health, i.e., free of disease and disability. DALYs are the sum of the years of life lost due to premature mortality (YLLs) and years lived with disability (YLDs). YLLs were calculated by multiplying the number of deaths at each age by a standard life expectancy at that age. The number of deaths at each age were calculated with use of the average European case-fatality rate of 11%; about 3% in-hospital and 8% out-of-hospital [2], [33]. To allow for international comparisons, the life expectancy was calculated using the Coale-Demeny model West life tables, with a life expectancy at birth of 80 years for males and 82.5 years for females [34].

YLDs were calculated in three steps [35]. First, data was gathered on the incidence, age and sex distribution of patients treated at the ED or hospitalized due to TBI. Second, the incidence data was divided into the injury categories “Concussion” and “Skull-brain injury” of the EUROCOST classification system [36]. Finally, the grouped incidence data was combined with the disability weights and durations developed within the framework of the European INTEGRIS (Integration of European Injury Statistics) study [35]. Registered cases were multiplied with the 1-year disability weight, the proportion of lifelong consequences (Concussion: 4% ED, 21% hospitalized; Skull-brain injury: 13% ED, 23% hospitalized) and the duration (life expectancy at age of injury, by sex). The mean 1-year disability weights included the temporary and lifelong consequences for cases seen in EDs and those recorded in hospital discharge registers for both concussions (Temporary: 0.015 ED, 0.100 hospitalized; Lifelong: 0.151) and skull-brain injuries (Temporary: 0.090 ED, 0.241 hospitalized; Lifelong: 0.323).To compare the impact of TBI with that of other injuries, YLDs for the other injuries were also calculated with disability weights obtained from the INTEGRIS study. The disability weights were derived from empirical follow-up data on the health-related quality of life of individual trauma patients, and adjusted for population norms, age and gender [35].

### Data and statistical analysis

All statistical analyses were carried out using the statistical package SPSS for Windows, version 21 (IBM SPSS Statistics, SPSS Inc, Chicago, IL). Descriptive statistics were used to provide insight in the characteristics of TBI patients. Continuous variables were described by presenting the median and interquartile range. Incidence rates per 100,000 person years were calculated using population data from Statistics Netherlands [37]. A value of p<0.05 was used to determine statistical significance. All data reported in this article are national estimates.

## Results

### Incidence

In the period 2010–2012, annually 34,681 patients visited the ED due to TBI (Table 2), comprising about 4% of the total injury-related ED visits per year in the Netherlands. The overall incidence rate of ED visits due to TBI was 213.6 per 100,000 person years, 241.9 for males and 175.3 for females respectively. Incidence rates were highest in children (268.2), young adults (271.6) and older patients in the age of 75–84 years (307.6) or 85 and older (578.2). The majority of patients sustained a TBI because of a home and leisure injury (47.9%) or traffic injury (33.5%).

**Table 2:** Incidence and characteristics of traumatic brain injuries in the Dutch population (2010–2012)^1^.

	Dutch Injury Surveillance System N = 3,762 (%)	National estimate N = 34,681 (%)	Incidence (per 100,000) Total: 213.6
**Gender**			
Male	2,162 (57.5)	19,937 (57.5)	241.9
Female	1,600 (42.5)	14,744 (42.5)	175.3
**Age**			
0–14	846 (22.5)	7,793 (22.5)	268.2
15–24	601 (16.0)	5,538 (16.0)	271.6
25–44	714 (19.0)	6,584 (19.0)	148.7
45–64	789 (21.0)	7,281 (21.0)	156.1
65–74	332 (8.8)	3,062 (8.8)	211.4
75–84	287 (7.6)	2,648 (7.6)	307.6
85+	192 (5.1)	1,775 (5.1)	578.2
**Accident category and type of road user**			
Home and leisure	1,806 (48.0)	16,628 (47.9)	
Traffic	1,256 (33.4)	11,616 (33.5)	
* Pedestrian*	*66 (5.3)*	*613 (5.3)*	
* Bicyclist*	*706 (56.9)*	*6,522 (56.9)*	
* Moped occupant*	*151 (12.2)*	*1,406 (12.3)*	
* Motor vehicle/scooter occupant*	*63 (5.1)*	*575 (5.0)*	
* Passenger vehicle occupant*	*205 (16.5)*	*1,898 (16.5)*	
* Other*	*49 (4.0)*	*455 (4.0)*	
* Unknown*	*16*	*148*	
Sport	307 (8.2)	2,824 (8.1)	
Occupational	109 (2.9)	1,003 (2.9)	
Assault	247 (6.6)	2,269 (6.5)	
Self-mutilation	18 (0.5)	171 (0.5)	
Other	19 (0.5)	172 (0.5)	
**Type of brain injury** 2 ^,^ 3			
* Concussion*		8,983 (44.7)	
* Fracture*			
Vault		317 (1.6)	
Base		1,319 (6.6)	
Other/unqualified		330 (1.6)	
Multiple fractures		130 (0.6)	
* Lesion*			
Cerebral laceration/contusion		1,977 (9.8)	
Subarachnoid/sub-/extradural hemorrhage		1,598 (7.9)	
Other/NFS intracranial hemorrhage		262 (1.3)	
Intracranial injury, other/NFS nature		5,116 (25.5)	
* Late effects*			
Musculoskeletal and connective tissue		46 (0.2)	
Nervous system		18 (0.1)	
* Nerve injury*			
Optic nerve and pathways		3 (<0.1)	
Unknown		14,581 (42.0)	
**Number of injuries**			
1 injury	1,065 (28.3)	9,766 (28.2)	
2 injuries	2,033 (54.0)	18,773 (54.1)	
≥3 injuries	664 (17.7)	6,142 (17.7)	
**Hospitalization**			
Not admitted	1,633 (43.5)	15,024 (43.4)	
Unknown	8	70	
1–3 days	1,424 (70.4)	13,146 (70.4)	
≥4 days	597 (29.6)	5,529 (29.6)	
N days unknown	106	982	

1 Mean number per year in the period 2010–2012.

2 Traumatic brain injury diagnoses (ICD-9 codes): *Concussion:* Concussion (850): *Cranial fracture:* Fracture of vault of skull (800); Fracture of base of skull (801); Other and unqualified skull fractures (803); Multiple fractures involving skull or face with other bones (804): *Lesion:* Cerebral laceration and contusion (851); Subarachnoid, subdural, and extradural hemorrhage after injury (852); Other and unspecified intracranial hemorrhage after injury (853); Intracranial injury of other and unspecified nature (854): *Late effects:* Late effects of musculoskeletal and connective tissue injuries (905); Late effects of injuries to the nervous system (907): *Nerve injury:* Injury to optic nerve and pathways (950): *Head injury, unspecified* (959, N = 0).

3 Data on injury type (ICD) only known for hospitalized patients in the LMR database (National estimate: N = 20,100).

Patients that sustained a TBI due to a traffic accident often concerned bicyclists (56.9%) and passenger vehicle occupants (16.5%). Home and leisure injuries often concerned a fall among 0–5-year-olds and elderly patients (aged 60 years and older). ED visits due to TBI often included the diagnoses concussion (44.7%), intracranial injury of other or unspecified nature (25.5%) and cerebral laceration or contusion (9.8%). Almost one in three TBI patients were treated for more than one injury and more than half of the patients were hospitalized, most frequently for 1 or 2 days (61.7% of the hospitalized patients).

### Cost-of-illness

The estimated total costs of TBI in the Netherlands was €314.6 million per year (Table 3). Total direct healthcare costs (€158.6 million) were comparable to indirect costs (€155.9 million), whereas in the working population per case mean direct healthcare costs were more than 3 times lower than the indirect costs. Overall, the mean total costs per case were €18,030, and were higher for men (€19,540) than for women (€14,940). This difference is mostly driven by the difference in indirect costs per TBI patient (males €15,416; females €10,257; p<0.001). The estimated total amount of omitted work days among TBI patients with paid employment was 44 days per case, and significantly differed between men (mean 46 days) and women (mean 38 days) (p<0.001). Both direct and indirect costs per TBI patient increased with the length of hospital stay.

**Table 3:** Cost-of-illness by hospitalization and gender (2010–2012).

	Hospitalization	Direct costs per case^1^	Indirect costs per case^1^	Total costs per case^1^	Total costs (€)
**Total**	0–7 days	3,584	12,454	16,040	234,259,230
	>7 days	9,854	21,431	31,280	64,608,290
	Total	**4,361**	**13,668**	**18,030**	**314,592,930**
**Men**	0–7 days	3,413	14,116	17,530	149,815,870
	>7 days	8,809	22,216	31,020	41,805,590
	Total	**4,128**	**15,416**	**19,540**	**202,953,300**
**Women**	0–7 days	3,812	9,479	13,290	84,443,360
	>7 days	11,433	18,638	30,070	22,802,690
	Total	**4,680**	**10,257**	**14,940**	**111,639,630**

1 Mean costs per case: indirect costs per case are presented as an average of only the working population (15 to 65 years).

**Figure 1:**
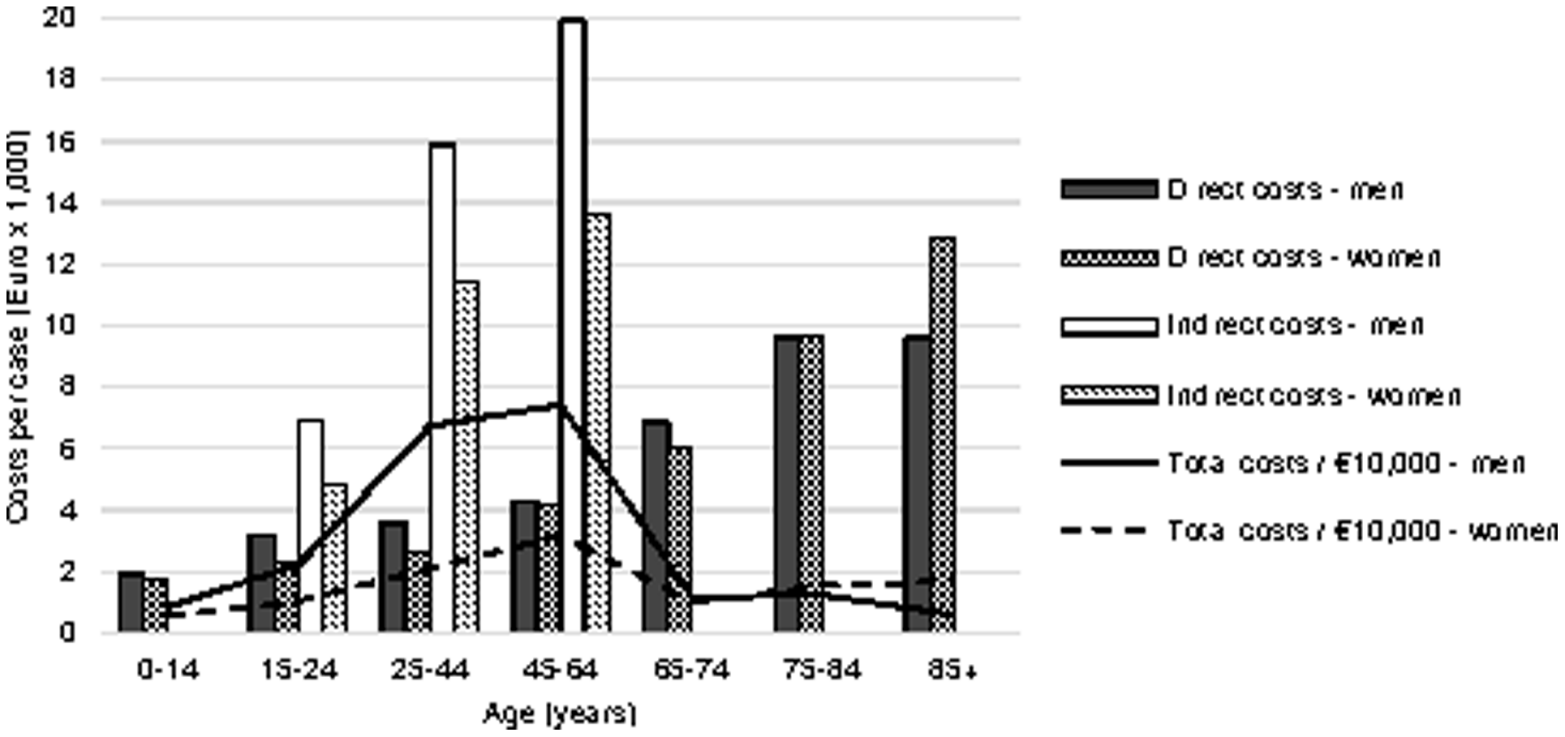
Mean direct and indirect costs per case and total costs by age and gender (2010–2012).

The average direct costs per case increased with age (Figure 1). Mean direct costs per case were higher (up to €950) for men than for women in the ages up to 74 years, while in individuals aged over 75 years women had much higher mean direct costs per case (up to € 3,210) than men. Indirect costs (applicable to individuals aged 15–64 years old) also increased with age, and were higher (up to €6,280) for men than for women.

### Disability-adjusted life years

**Figure 2:**
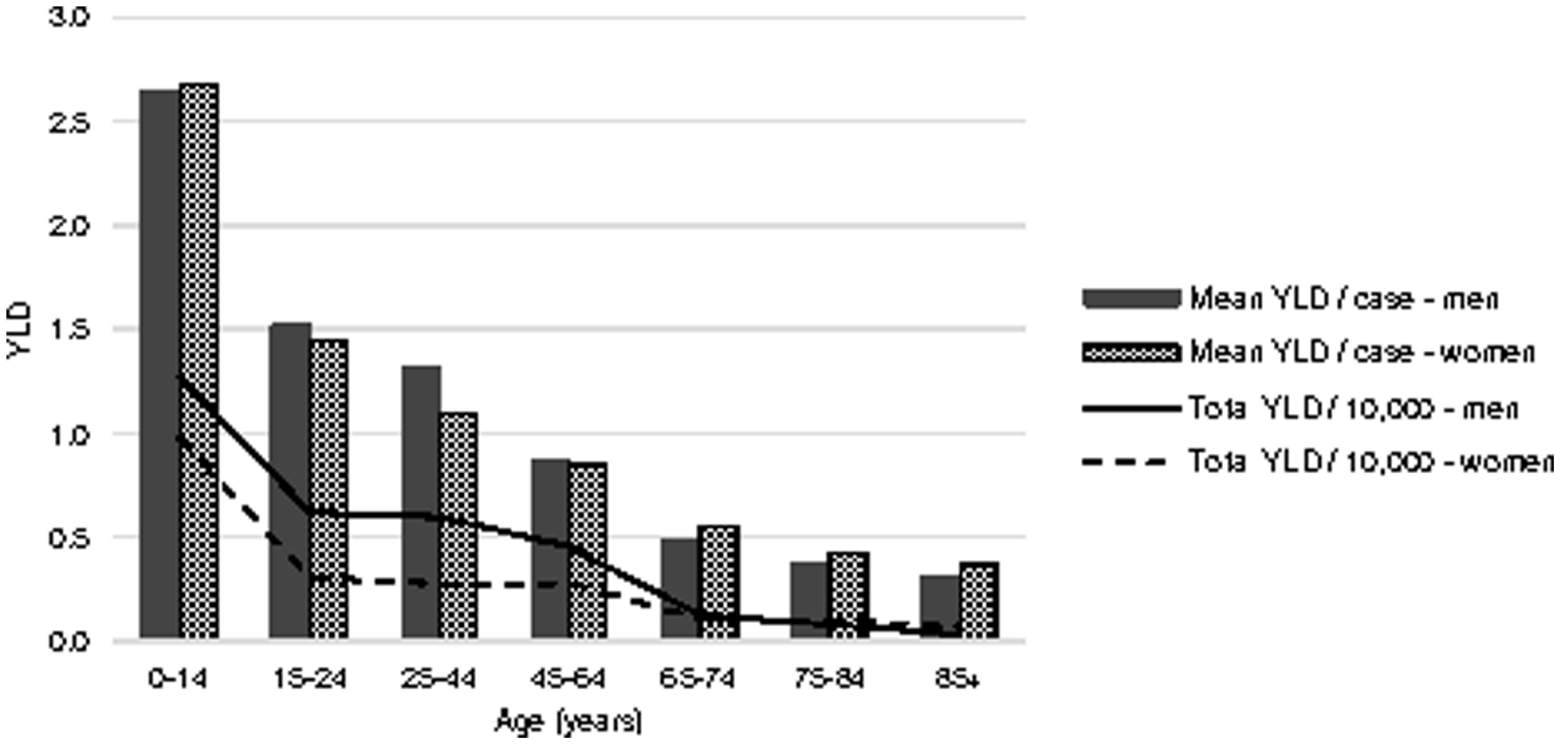
Mean years lived with disability by age and gender (2010–2012). YLD: years lived with disability.

TBI resulted in 52,998 YLD and 118,207 YLL respectively, amounting to 171,205 DALYs (on average 7.07 DALYs per TBI patient, Table 4). Overall, 69% of the total burden was caused by premature mortality. The burden due to permanent (lifelong) disability was high compared with temporary (short-term) disability. Men were responsible for 59% of the total burden of TBI, and had higher YLDs, YLLs and DALYs per case than women (YLD per case: 2.29 in men vs 2.05 in women; YLL per case: 4.97 vs 4.76; DALY per case: 7.27 vs 6.81). Mean YLD decreased with age in both men and women, and was highest among 0–14-years-olds (Figure 2).

**Table 4:** Total temporary and lifelong years lived with disability, years of life lost and disability-adjusted life-years per 1-year interval (2010–2012).

		YLD ED visits		YLD hospital admission				
	N	Temporary	Lifelong	Temporary	Lifelong	YLL	Total DALYs^1^	DALYs per case
Men	13,877	56	1,077	2,098	28,603	69,022	100,856	7.27
Women	10,330	47	941	1,470	18,706	49,185	70,348	6.81
Concussion	12,580	54	1,023	897	13,540	118,207	171,205	7.07
Skull-brain injury	11,631	50	995	2,670	33,769			
**Total**	**24,211**	**104**	**2,018**	**3,567**	**47,309**	**118,207**	**171,205**	**7.07**

1 Disability-adjusted life-years (DALYs) per year.

YLD: years lived with disability; ED: emergency department; YLL: years of life lost.

### TBI in comparison to other injury categories

In the period 2007–2011, TBI accounted 10% of the total YLDs and 12% of the lifelong YLDs caused by all injuries in the Netherlands (data not shown). Concussion and skull-brain injury both were ranked in the top 5 of injuries with highest total YLDs, after fractures of the knee or lower leg, ankle, and foot or toes (Table 5). Skull-brain injury accounted for the highest YLDs per case after spinal cord injury: 2.89 and 14.68 respectively (data not shown).

**Table 5:** Top ten injuries with highest disability in the Netherlands by accident category (2007–2011)^1^.

Rank	Home and leisure	Traffic	Sport	Occupational	Total
1	Fracture ankle	Fracture knee/lower leg	Fracture knee/lower leg	Fracture foot/toes	Fracture knee/lower leg
2	Fracture foot/toes	**Skull-brain injury**	Fracture ankle	Fracture knee/lower leg	Fracture ankle
3	Fracture knee/lower leg	**Concussion**	Fracture foot/toes	Fracture ankle	Fracture foot/toes
**4**	**Concussion**	Fracture ankle	Lux/dist ankle/foot	Spinal cord injury	**Skull-brain injury**
**5**	**Skull-brain injury**	Spinal cord injury	Lux/dist knee	**Skull-brain injury**	**Concussion**
6	Hip fracture	Fracture foot/toes	**Concussion**	Complex soft tissue arm/hand	Spinal cord injury
7	Spinal cord injury	Hip fracture	Fracture wrist	Lux/dist ankle/foot	Hip fracture
8	Fracture upper arm	Fracture shoulder	**Skull-brain injury**	**Concussion**	Lux/dist ankle/foot
9	Lux/dist ankle/foot	Fracture upper arm	Fracture upper arm	Lux/dist knee	Fracture upper arm
10	Fracture wrist	Fracture upper leg	Fracture shoulder	Open wound	Lux/dist knee

1 Ranked by total years lived with disability (YLD) for short- and long-term disability.

Lux/dist: luxation/distortion.

## Discussion

The purpose of this paper was to estimate the incidence, cost-of-illness and disability-adjusted life years (DALYs) of TBI in the Netherlands. Our study revealed that TBI imposes a substantial economic and disease burden (on average 7.1 DALYs per TBI patient) on the Dutch population, accounting for more than 4% of injury-related ED visits, 9% of the injury-related costs and 10% of the injury-related YLDs in the Netherlands.

**Figure 3:**
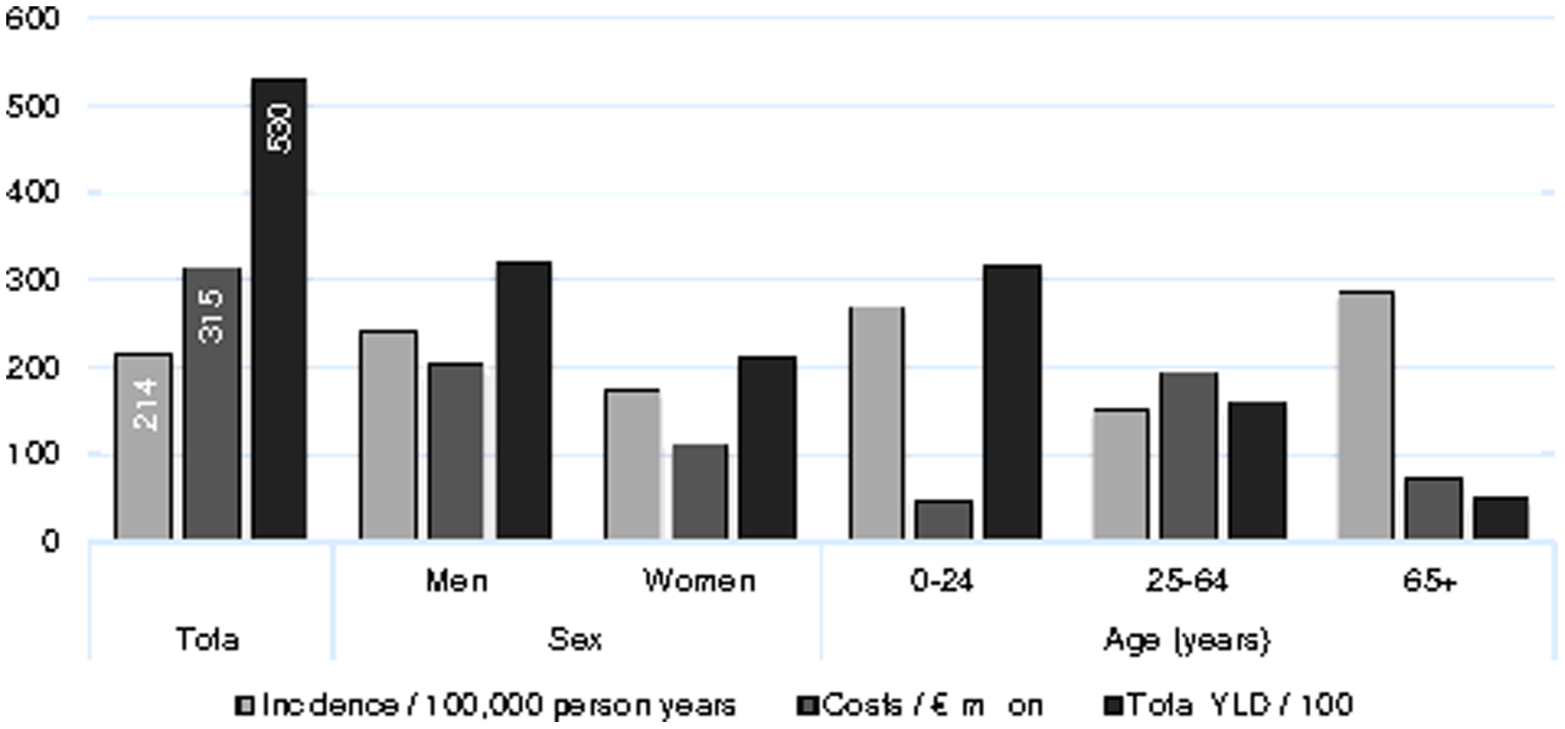
Economic and disease burden of traumatic brain injury in the Netherlands (2010–2012). YLD: years lived with disability.

The integrated approach of our study showed that the incidence and burden of disease among children and young adults aged 0–24 years is high, whereas the economic consequences for this group were low due to relatively shorter hospitalization and almost no indirect costs (Figure 3). The reverse is shown in the 25–64-year-olds, who have relatively low incidence and high economic costs, driven by loss of productivity. Older patients aged 65+ had highest incidence of TBI, leading to considerable direct healthcare costs, and a relatively low disease burden.

### Comparison of results to other studies

#### Incidence

Our estimated incidence rate of ED treated, hospitalized and fatal TBI for the Netherlands of about 214 per 100,000 person years was lower than the estimated rate of hospitalized and fatal TBI for Europe of about 235 per 100,000 persons years [2]. This difference may partly be explained by the time period covered in the studies. The European rate was derived from studies with data over 1974 to 2000, with incidence rates ranging from 150 to 300 per 100,000 person years [2], whereas our study included data from 2010 to 2012. Compared to the US, the Dutch estimated incidence rates of ED visits, hospital admissions and deaths are considerably lower. It is estimated that the incidence of TBI in the US is 577 per 100,000 person years in 2006 [38], comprising about 1,365,000 ED visits (81%), 275,000 hospitalizations (16%) and 52,000 deaths (3%). However, other population-based studies suggest that the incidence of TBI in the US is somewhat lower, between 180 to 250 per 100,000 person years in 1965 to 1996 [2], [11].

Consistent with prior research [2], [38], TBI incidence was higher among men than women, and highest among children and older people. Whereas motor vehicle accidents and falls were the most common mechanisms of injury in previous studies in Europe [2] and the US [6], [17], [38], our sample showed a high number of ED treatments among bicyclists in the traffic setting. Cycling is a very popular form of transport and recreation in the Netherlands, as up to 28% of all trips nationwide are made by bicycle [39]. The popularity of cycling however also imposes a high burden on society, due the large number of (brain) injuries among cyclist [40]–[42]. Bicycle helmets are not compulsory in the Netherlands and are only commonly used among road cyclist, mountain bikers and young children.

#### Cost-of-illness

TBI accounted for 9% of total costs of all injuries in the Netherlands (about €3.5 billion). The direct healthcare costs of TBI are on average €4,300 per case. This is in line with the outcomes of a previous study on the costs of all types of injuries in the Netherlands in 2004 that estimated the average direct healthcare costs of skull and brain injury cases at €3,100 [12]. This is approximately €4,100 when converting 2004 Euros to 2012 Euros using consumer price index.

Compared to other European countries our estimation of direct healthcare costs of TBI are somewhat higher [5]: from €2,700 in whole Europe, to €2,930 in Germany, €3,490 in Spain and €3,453 in Sweden after adjustment for inflation up to 2012. Estimates from the US are however more than two times the estimates in our present study: about €23,500 acute hospital charges per TBI [15], €6,200 per TBI in Missouri [17], and €8,500 to €35,000 for mild to severe hospitalized patients [6] - all scaled to 2012 price levels and 2012 Euro. These differences can partly be explained by differences in cost calculations. The European cost calculations were limited to inpatient costs while the current study included also extramural healthcare costs, and most US studies used charges instead of unit costs. Although the methodology of cost calculations varied considerably, our study confirms that indirect costs of TBI are far higher than direct healthcare costs of TBI [9], [17], [43], [44], costs of TBI are higher among men than women and increase with age [5] and that the costs increase with the length of hospital stay [6]. The latter suggests that the economic burden of TBI varies considerably by TBI severity.

Overall, TBI imposes a high economic burden on society and, together with hip fracture, is a leading source of hospital costs [13] and direct healthcare costs [12] in the Netherlands due to high healthcare costs per patient.

#### Disability-adjusted life years

TBI accounted for 10% of total YLD and 12% of the lifelong YLD caused by all injuries in the Netherlands, due to lifelong consequences in a relative young patient group. TBI resulted in both high temporary and lifelong YLD among road traffic injuries and home and leisure injuries, as confirmed in the literature [19], [45]. TBI is one of the leading causes of disease burden compared to other injuries and diseases in the Netherlands. TBI imposes a disease burden comparable to that of depression, diabetes, and lung cancer, which are all in the top 10 diseases with highest total DALY in the Netherlands [46]. Mean YLD decreased with age and was highest among children (0–14 years). This can partly be explained by the use of the expected number of years of life remaining as the duration of TBI in the YLD calculation. This method assumes that a proportion of the TBI patients will live with disability outcomes for the remainder of their expected lifetime. Therefore the duration used in the YLD calculation equaled the life expectancy at age based on the Coale-Demeny model West life tables [34]; in our sample on average 45 years in men and 43 in women. This may have led to a higher estimate of the years lived with disability after TBI in comparison to the use of a fixed average duration for TBI.

### Limitations

The number of deaths due to TBI in the Netherlands could not be generated from national death statistics, because these are only available for specific diseases (e.g., type of cancer, cardiovascular diseases) or injuries specified by cause (e.g., traffic accidents, falls, drowning, self-mutilation). Therefore, the YLL component of the total DALY was estimated with use of the European average case fatality rate of TBI, derived from 18 studies. This overall case fatality rate was on average about 11 per 100 persons with TBI; about 3% in-hospital and 8% out-of-hospital deaths among patients with TBI [2], [33]. Due to the use of the average European overall case fatality rate, the number of YLLs and thereby the disease burden of TBI may be over- or underestimated. However, actual case fatality rates and disease burden of TBI may be even higher due to higher excess mortality in the long-term [47]–[49]. In order to improve the YLL and disease burden estimates of TBI and other injuries and diseases in the Netherlands, specific (long-term) mortality data should be registered and available for future research.

Other limitations concern the classification of TBI and the calculation of costs. TBI patients treated at the ED were registered as having a “Concussion” or “Other skull – brain injury”. No additional data was available on ICD-codes, AIS-codes or a Glasgow Outcome Scale to uniformly determine TBI; data that was available for the majority of the hospitalized patients.

The ICD-9 codes used to determine the type of TBI among hospitalized patients slightly differed from those recommended by the Center for Disease Control (CDC) [50], in that this study also included the late effects of TBI (ICD-9 codes 905, 907, 950). These late effects however comprise far less than 1% of all hospitalized traumatic brain injuries in the Netherlands, and therefore will not complicate comparison of our results to those of other studies in which the CDC ICD-9 codes for TBI were used.

The cost-of-illness of TBI may have been overestimated because of the use of a patient follow-up survey to obtain information on healthcare consumption and labor status. Comparison of the hospital discharge data and the patient follow-up data indicated that there is a higher response among the more severe injured patients. This may lead to an overestimation of the costs and disease burden of TBI.

On the other hand, our estimation of indirect costs of TBI comprised only costs of lost work productivity for TBI patients of working age. Other potential sources of indirect costs, such as the work productivity and finances of families and caregivers were not incorporated in this study. Previous research showed that TBI imposes a significant level of financial burden on families and caregivers [51], [52], which is directly related to the severity of TBI [51]. Total indirect costs of TBI will therefore be far higher than estimated in this study, particularly among children and elderly with caregivers in the working age.

Our study is limited to TBI patients that were treated at the ED or admitted to hospital. Patients who consulted a GP were not included in our overview. Hence, incidence rates, cost-of-illness and burden of TBI may be even higher [53]. According to registries from Dutch general practice, in 2012 about 7,600 persons contacted their General Practitioner (GP) or after-hours General Practitioner Co-operation (GPC) due to TBI [54]. Assuming that direct healthcare costs of GP visits are on average €39 per contact (Table 1; mean costs for practice or telephone consultation, and home visit), and the indirect costs and disease burden of TBI will not be larger than that of ED-treated patients, they will add about 7–8% only to our cost estimate and about 1–2% only to our DALY estimate.

### Recommendation for future research

The results of our study reveal that TBI imposes a relatively high economic and health impact compared to all injuries and diseases in the Netherlands. TBI is a growing worldwide problem, as recent reports suggest a rapid increase in ED visits and hospitalizations resulting from traumatic brain injury, especially fall-related TBI in older adults [55]–[57] and traffic-related TBI [58], [59]. There is a need for prevention programs targeting on the reduction of incidence and severity of TBI. On the bases of our study, we conclude that especially children and young adults aged 0–24 years, men aged 25–64 years and traffic injury victims (in the Netherlands especially bicyclists) and home and leisure injury victims are an important target for intervention. In the working population, screening for risk of problems to return to work and immediate rehabilitation after TBI may help to minimize lost productivity [60].

Future research should examine how helmet use among cyclists can be increased. Bicycle helmets have shown to be highly effective in preventing head, brain, and facial injuries to cyclist [61], [62]. Previous research in Canada showed that helmet legislation may be an effective tool in the prevention of childhood bicycle-related head injuries [63]. Overall, future research on the population impact of TBI in terms of costs and disease burden should also include patients who receive no treatment or out-of-hospital treatment (e.g., from a GP or by sports trainers).

### Conclusions

This study provided comprehensive population-based estimates on the epidemiology, costs and disease burden (in DALYs) of ED-treated and hospitalized persons with TBI over 2010–2012 in the Netherlands. The study included all age groups, all TBI severities, and both patients treated at the ED and hospitalized patients. The economic and health consequences of TBI are substantial. Prevention programs are needed to reduce incidence and severity of TBI. The integrated approach of assessment of incidence, costs and disease burden (in DALYs) of TBI enables the detection of all important risk groups in TBI.
